# Appendicular skeletal muscle mass and quality estimated by bioelectrical impedance analysis in the assessment of frailty syndrome risk in older individuals

**DOI:** 10.1007/s40520-021-01879-y

**Published:** 2021-06-12

**Authors:** Małgorzata Kołodziej, Anna Sebastjan, Zofia Ignasiak

**Affiliations:** Department of Biostructure, University School of Physical Education in Wroclaw, Al. I. J. Paderewskiego 35, 51-612 Wroclaw, Poland

**Keywords:** Appendicular skeletal muscle, Bioelectrical impedance analysis, Frailty, Muscle quality, Healthy aging

## Abstract

**Background and aim:**

The rising aging index of many populations necessitates the continuous evolution of geriatric assessment methods, especially the ones used to identify frailty and the risk of frailty. An appropriately early diagnosis of adverse changes in skeletal muscles can reduce the risk of functional limitations in elderly persons. The aim of this study was to assess the correlation between the appendicular skeletal muscle mass and quality, estimated by the bioelectrical impedance analysis method, and the risk of prevalence of the pre-frailty state in elderly persons.

**Methods:**

One-thousand-and-fifteen subjectively healthy persons aged 60–87 years were tested. Anthropometric measurements and physical fitness and activity measurements were carried out and the frailty phenotype was evaluated. Appendicular skeletal muscle mass was estimated using the bioelectrical impedance analysis method. Muscle quality was assessed through an index correcting strength relative to muscle mass and through the impedance phase angle. The correlation between the muscle mass and quality estimating parameters and the probability of identifying pre-frailty was checked using multiple logistic regression.

**Results:**

The prevalence of pre-frailty was 38%. The pre-frail persons were found to have a significantly lower muscle mass and quality than the non-frail persons, with the difference in the case of the muscle quality index nearly twice larger than for the muscle mass index. A significant logit model was obtained for pre-frailty prevalence, which was strongly dependent on the appendicular skeletal muscle mass (adjusted odds ratio (OR): 0.43, 95% CI 0.36–0.52, *p* < 0.001) and functional quality (adjusted OR: 0.26, 95% CI 0.18–0.38, *p* < 0.001) and less on age (adjusted OR: 1.10, 95% CI 1.07–1.13, *p* < 0.001).

**Conclusion:**

The strong correlation between the frailty phenotype and appendicular skeletal muscle mass and functional quality suggests that the two variables should be included in routine geriatric assessment with regard to frailty.

## Introduction

The aging of populations cannot be stopped, but according to the idea of “healthy aging”, its rate can be slowed down through diagnostics and appropriately early interventions, whereby the risk of early onset of chronic health problems, frailty, disability or death can be reduced [1].

Of particular importance for the functionality of older persons are the changes in their muscle mass and strength with age. A reduction in strength and muscle mass destabilizes the work of muscles, resulting in the deterioration in general physical fitness, an increased risk of falls and limitations in the performance of everyday activities. An intensified complex of these symptoms in elderly persons is most often characteristic of sarcopenia and/or the frailty syndrome [2–5].

Frailty is a multifaceted clinical syndrome considered in the three main aspects: physical, psychological and social. Physical frailty is characterized by a cumulative reduction in physical functionality and a susceptibility to adverse effects in physical stress conditions, such as illness or hospitalization. The frailty phenotype increases the risk of falling ill with several acute and chronic diseases, and also the risk of death. Frail elderly persons are exposed to sarcopenia, cachexia and wasting conditions [4–6].

Frailty can be assessed by various methods. The clinical assessment standard recommended by the Task Group of the International Conference of Frailty and Sarcopenia Research [3] is the highly confirmed frailty phenotype (FP) described by Fried et al. in 2001 [4]. Also Rockwood’s Clinical Frailty Scale (CFS), the FRAIL scale of the International Association of Nutrition and Ageing (IANA) and the Edmonton Frailty Scale (EFS) are recommended for screening examinations [3].

Body mass loss in elderly persons is the principal component taken into account by most of the frailty state identifying methods. However, there are reports that obesity may be an important determinant of frailty in old age [7, 8]. Therefore, the mass and quality of the skeletal muscles (considering their role in body functioning) seem to be more precise indicators of physical frailty than weight loss.

Among the many methods of assessing skeletal muscle mass in clinical practice and screening examinations, the bioelectrical impedance analysis (BIA) method is quite often used. The accuracy of the muscle mass estimates by the BIA method has not been clearly confirmed, therefore, the European Working Group on Sarcopenia in Older People (EWGSOP2) recommends using of raw impedance measures along with the Sergi et al. [9] equation for elderly persons [2]. This method is regarded as highly promising owing to the possibility of analyzing, in a wide frequency spectrum, such impedance components as: resistance, reactance and phase angle as identifiers of the condition of tissues [2, 10, 11]. A particular importance, although not yet completely explained, is attributed to the impedance phase angle. It is regarded as indicator of muscle cells quality, as it depends on their number and size and the integrity of cell membranes [10, 12].

The aim of our study was to assess the correlation between appendicular skeletal muscle mass and quality and the prevalence of frailty in elderly persons. We undertook an attempt to determine whether skeletal muscle mass and quality assessed on the basis of BIA measurements may be significant indicators of the risk of frailty syndrome prevalence in elderly persons.

## Materials and methods

### Study and its participants

In the years 2009–2015, thousand-and-sixteen persons (261 men and 755 women) aged 60–87 years (67.2 ± 5.5 years), who volunteered for the free tests, thanks to advertisements placed in local media and invitations sent to health centers and associations of elderly persons in the south-western areas of Poland, were tested. The pre-condition for inclusion in the tests was the age of 60+ years, no medical contraindications and independence and autonomy in everyday life. Participants were assessed as subjectively healthy on the basis of declarations of good health, no difficulty walking, and no limitations in daily activities. The study protocol was approved (18 February 2009) by the Senate Research Ethics Committee of the University School of Physical Education in Wroclaw. The research was carried out consistently with the Declaration of Helsinki recommendations in the Biokinetics Laboratory of the University School of Physical Education in Wrocław, certified according to the PN-EN ISO 9001:2009 Quality Management System (Certificate No. PW-48606-10E). The project was funded (Grant No. N404 075337) by the Ministry of Science and Higher Education. The participants were informed about the aim and methods of the study, the procedures used and the experimental risk. All the persons who declared their participation in the study signed a document of voluntary and informed consent.

### Bioelectrical impedance analysis and anthropometric measurements

Body height (Ht) and mass (Wt) were measured with an accuracy of, respectively, 0.1 cm and 0.1 kg by means of an electronic scale with an integrated SECA 764 digital stadiometer (certificate 93/42 EEC, manufacturer: Seca GmbH & Co. KG. Germany). Body composition, including skeletal muscle mass, was assessed using bioelectrical impedance analysis (BIA) by means of an 8-electrode TANITA MC 180 MA multi-frequency analyser (certificate 93/42 EEC, manufacturer: Tanita Corporation, Japan). The analyser measures impedance with an accuracy of 0.01 Ω and phase angle with an accuracy of 0.01°. Resistance, reactance and phase angle values were measured at the 50 kHz operating frequency of the 0.8 μA current. The measurement was performed in standing position on a platform with built-in four electrodes (2 per foot) and with two two-electrode handgrips enabling additional segmental readings separately for each limb and the trunk. Every day prior to the tests, proper repeatability of impedance measurement results was checked through two successive tests carried out on two volunteers. The analyser software uses proprietary equations for estimating fat-free mass and intra- and extracellular water contents in the body, which due to commercial sensitivity are unavailable for publication.

BIA measurements were carried out in the mornings, using the procedures indicated by the analyser manufacturer [13]. The subjects were asked not to eat, not to drink and not to undertake any physical activity at least 3 h before the test and to void the bladder immediately before the measurement. The presence of an electronic implant (e.g. a pacemaker), a body mass index of above 50 kg/m^2^ and a limb amputation were the contraindications to measurements by the BIA method.

In compliance with the latest EWGSOP2 recommendations [2], appendicular skeletal muscle mass (ASMM) was estimated using the predictive equation published by Sergi et al. [9]:

$$ {\text{ASMM}}\,({\text{kg}}) = - 3.964 + (0.227*{\text{Ht}}^{2} /R) + (0.095*{\text{Wt}}) + (1.384*{\text{sex}}) + (0.064*X_{c} ), $$

ASMM: appendicular skeletal muscle mass; Ht: height (cm); *R*: resistance (Ω); Ht^2^/*R*: resistance index (cm^2^/Ω); Wt: weight (kg); sex: men = 1 and women = 0; *X*_*c*_: reactance (Ω).

To minimize the differences stemming from inter-subject variability and considering the strong correlation between muscle mass and body size, the ASMM value was adjusted to the square of body height [2].

### Evaluation of frailty phenotype (FP) criteria

Hand grip strength (HGS) was measured with an accuracy of 1 kg by means of a JAMAR (Sammons Preston Rolyan, USA) hydraulic hand dynamometer with an adjustable handle set to position 2. The recommendations of the American Society of Hand Therapists (ASHT) were adopted [14]. The subjects were asked to perform two maximum grip strength tests for alternately the left hand and the right hand. Each of the tests lasted 3–5 s and the inter-measurement interval was 15–20 s long. The highest value from all the tests was recorded as the HGS value. The frailty criterion for HGS was the cut-off values proposed by Fried, with sex and body mass index taken into account: HGS ≤ 29 kg at BMI ≤ 24 kg/m^2^, HGS ≤ 30 kg at BMI 24.1–28 kg/m^2^ and HGS ≤ 32 kg at BMI > 28 kg/m^2^ for the men and HGS ≤ 17 kg at BMI ≤ 23 kg/m^2^, HGS ≤ 17.3 kg at BMI 23.1–26 kg/m^2^, HGS ≤ 18 kg at BMI 26.1–29 kg/m^2^ and HGS ≤ 21 kg at BMI > 29 kg/m^2^ for the women [4].

The hand-grip strength to estimated appendicular skeletal muscle mass ratio (HGS/ASMM) was adopted as the muscle functional quality index [2, 15].

Walking speed was assessed using the 8-foot-up-and-go test from the Rikli & Jones Senior Fitness Test Kit [16]. The number of seconds needed to get up from a seated position, walk 8 feet, turn and return to the seated position was measured (total distance was 16 feet). It was recommended to cover the distance as quickly as possible. The critical time to walk 16-feet was adjusted to the Fried criteria for the time in the 15-feet walk test [4] (critical time for 16-feet = critical time for 15-feet*16/15). Considering sex and body height, the frailty criterion was satisfied when test time ≥ 7.5 s (at Ht ≤ 173 cm) and ≥ 6.4 s (at Ht > 173 cm) for the men and ≥ 7.5 s (at Ht ≤ 159 cm) and ≥ 6.4 s (at Ht > 159 cm) for the women.

Weekly physical activity (PA) was evaluated using the International Physical Activity Questionnaire (IPAQ) [17]. The participants would answer questions concerning the frequency and duration of their low-, moderate- and intensive-level physical activities. PA values < 383 kcal/week for the men and PA < 270 kcal/week for the women were adopted as indicative of frailty criterion satisfaction [4].

Applying the criteria of the frailty phenotype model proposed by Fried et al. [4], two groups: a non-frailty group (*n* = 630), satisfying none of the frailty criteria, and a pre-frailty group (*n* = 385), satisfying one or two criteria, were distinguished (more details can be found in Ignasiak et al. [18]). Frail cases were excluded from the study because the state of frailty was identified only in one person (at least three frailty criteria were satisfied).

### Statistical analysis

The normality of the distribution of all the variables was tested using the Shapiro–Wilk test. No normal distribution was confirmed for most of the variables, but the low asymmetry of the distributions and the possibility of comparing the results with the results reported by other authors induced us to use classical statistical description measures. The results were presented as mean ± standard deviation (Mean ± SD) and 95% confidence levels were calculated for the mean (95% CI).

Non-parametric Kruskal–Wallis test was used to evaluate the differences between the gender and frailty groups. The differences between the non-frail persons and the pre-frail persons in the gender groups were verified using the *U* Mann–Whitney test. The probability of pre-frailty state (pre-frail = 1; non-frail = 0) identification was assessed using logistic regression. Skeletal muscle mass and quality indices, gender (men = 1, women = 0), age and phase angle were included as independent variables. No *X*_*c*_ or *R* was included since they are trigonometric functions of the phase angle and are variables in the BIA equation for ASMM. The variables which constituted the frailty criterion (HGS, Walking time and PA) in our study were also not taken into account. The statistical significance of individual regression coefficients was tested using Wald Chi-square statistics. The variables which had been found to be significantly correlated with pre-frailty in the univariate analyses were taken into account in the multiple logistic regression (multivariable analysis). Using the reverse stepwise technique, all selected factors were initially entered into the model and then removed if the *p* value for Wald’s test exceeded 0.05. Goodness-of-fit was checked with the Hosmer–Lemeshow test and was accepted at *p* > 0.05. All the analyses were carried out using STATISTICA 13.1 (StatSoft Polska S.A.). The statistical significance of the results was accepted at *p* < 0.05.

## Results

The descriptive characteristics of the subjects and the differences between the non-frail persons and the pre-frail persons are presented in Table 1. One patient was frail and was excluded from the analysis. For all variables significant differences were found between the gender groups (*p* < 0.05 for PA and *p* < 0.01 for all the other variables). As expected, in comparison with the women, the men were characterized by greater body mass (Wt) and height (Ht), appendicular skeletal muscle mass (ASMM) and hand-grip strength (HGS). The women were characterized by higher resistance (*R*) and reactance (*X*_*c*_) values, a smaller phase angle (PhA) and a lower walking speed than the men. The higher (by over 17%) proportion of skeletal muscles (ASMM/Ht^2^) in the men than in the women generated significantly greater (by over 16%) grip strength (corrected for ASMM) in the former. The results of the tests of the significance of the differences between the women and the men, which had been expected and repeatedly reported in the literature, were not included in the table.

**Table 1:** Descriptive characteristics of study participants

	Men			Women		
	Non-frail (*n* = 207)	Pre-frail (*n* = 54)	*p*	Non-frail (*n* = 423)	Pre-frail (*n* = 331)	*p*
	Mean ± SD (95% CI)	Mean ± SD (95% CI)		Mean ± SD (95% CI)	Mean ± SD (95% CI)	
Age (years)	67.8 ± 5.3 (67.1–68.5)	70.9 ± 6.1 (69.3–72.6)	0.001	65.6 ± 4.6 (65.1–66.0)	68.3 ± 5.8 (67.7–68.9)	< 0.001
Height (cm)	172.8 ± 5.9 (172.0–173.6)	172.2 ± 6.2 (170.5–173.9)	0.749	158.7 ± 5.5 (158.2–159.3)	158.3 ± 6.2 (157.6–159.0)	0.187
Weight (kg)	87.3 ± 12.2 (85.7–89.0)	79.8 ± 12.1 (76.5–83.1)	< 0.001	71.7 ± 13.0 (70.4–72.9)	69.9 ± 11.0 (68.7–71.1)	0.246
BMI (kg/m^2^)	29.2 ± 3.5 (28.7–29.7)	26.9 ± 3.6 (25.9–27.8)	< 0.001	28.5 ± 5.2 (28.0–29.0)	27.9 ± 3.9 (27.4–28.3)	0.821
HGS (kg)	45.9 ± 8.5 (44.8–47.1)	37.6 ± 9.4 (35.1–40.2)	< 0.001	27.1 ± 5.2 (26.6–27.6)	24.1 ± 7.7 (23.3–25.0)	< 0.001
Walking time 15 feet(s)	4.98 ± 0.59 (4.90–5.06)	6.10 ± 1.00 (5.82–6.37)	< 0.001	5.29 ± 0.59 (5.23–5.34)	6.79 ± 0.80 (6.71–6.88)	< 0.001
PA (kcal/week)	445 ± 149 (419–471)	348 ± 132 (304–392)	0.002	437 ± 156 (414–461)	367 ± 170 (344–390)	< 0.001
*R* (Ω)	497.1 ± 48.9 (490.4–503.8)	530.9 ± 61.2 (514.2–547.6)	< 0.001	613.8 ± 72.8 (606.9–620.8)	663.6 ± 67.4 (656.3–670.9)	< 0.001
*X*_*c*_ (Ω)	50.3 ± 7.6 (49.2–51.3)	51.9 ± 9.6 (49.3–54.5)	0.424	56.7 ± 8.5 (55.9–57.5)	57.2 ± 9.7 (55.9–58.4)	0.177
PhA ( °)	5.77 ± 0.59 (5.69–5.85)	5.57 ± 0.74 (5.37–5.77)	0.045	5.29 ± 0.59 (5.23–5.35)	5.18 ± 0.57 (5.12–5.24)	0.002
ASMM (kg)	22.7 ± 2.5 (22.4–23.1)	21.2 ± 2.2 (20.5–21.8)	< 0.001	15.9 ± 2.1 (15.7–16.1)	15.2 ± 1.8 (15.0–15.4)	< 0.001
ASMM/Ht^2^ (kg/m^2^)	7.60 ± 0.64 (7.52–7.69)	7.13 ± 0.66 (6.95–7.31)	< 0.001	6.32 ± 0.79 (6.25–6.40)	6.06 ± 0.58 (5.99–6.12)	< 0.001
HGS/ASMM	2.03 ± 0.38 (1.98–2.08)	1.77 ± 0.37 (1.67–1.87)	< 0.001	1.72 ± 0.35 (1.69–1.75)	1.59 ± 0.48 (1.54–1.64)	< 0.001

*SD* standard deviation, *95% CI* confidence interval, *BMI* body mass index, *HGS* hand-grip strength, PA physical activity, *R* resistance, *X*_*c*_ reactance, *PhA* phase angle, *ASMM* appendicular skeletal muscle mass, *Ht* height, *HGS/ASMM* muscle quality index

**Fig. 1:**
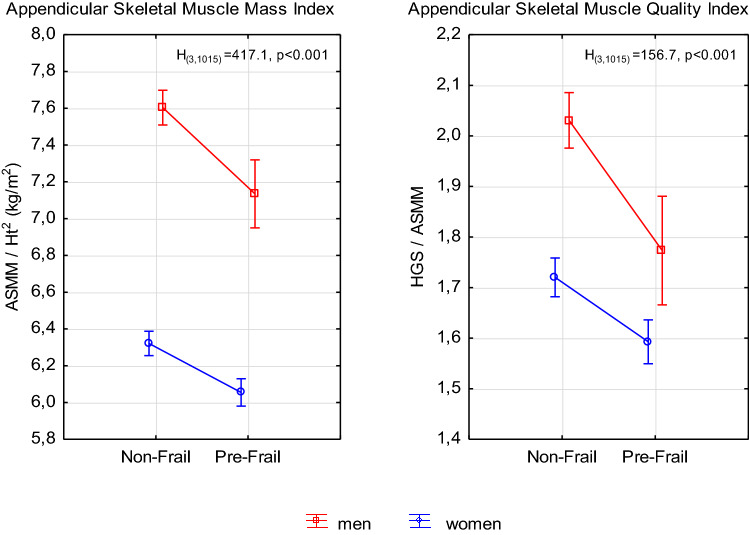
Differences in mean skeletal muscle mass and quality indices between groups of frailty status and age. Points with vertical segments stand for mean with 95% confidence level. *ASMM* appendicular skeletal muscle mass, *Ht* height, *HGS* hand-grip strength. *H*_(3,1015)_, *p*: Kruskal–Wallis rank test results

Of all the subjects, 38% were identified as pre-frail. In the case of the women (44%), the prevalence of pre-frailty was twice higher (*χ*^2^ = 44.36, *p* < 0.001) than in the men (21%). For both the frailty phenotype (FP) groups, the BMI indicated overweight in nearly 50% of the subjects and obesity in 30% of them. Besides the expected differences in age, mass and physical functionality parameters (i.e. strength, walking speed and physical activity), it was found that the pre-frail persons had significantly lower phase angle values than the non-frail persons (Table 1). The estimated ASMM in the pre-frail persons was significantly lower—by 7% in the men and by 4% in women—than in the non-frail persons. The differences between the pre-frail persons and the non-frail persons were nearly twice higher for the muscle quality index (HGS/ASMM)—by 13% in the men and 8% in the women (Fig. 1).

Univariate logistic regression was used to preliminarily check the correlation between the selected variables (without their mutual interaction) and the probability of identifying pre-frailty. A positive correlation of the pre-frail state with age and negative correlations with gender, phase angle and with the appendicular skeletal muscle mass and quality indices were confirmed (Table 2). Gender and the functional quality of the skeletal muscles most strongly affected the odds of pre-frailty. For men, the odds of pre-frailty were three times lower than the odds of the prevalence of this state in the women (OR = 0.33, 95% CI 0.24–0.46, *p* < 0.001). A similar reduction in the odds was observed in the case of the unit improvement in muscle quality (OR = 0.29, 95% CI 0.20–0.40, *p* < 0.001).

**Table 2:** Logistic regression models for probability of pre-frailty state in elderly persons

Predictor	Coefficient estimate	SE	OR (95% CI)	Wald statistic	*p* value
Univariate models					
Sex	− 1.10	0.17	0.33 (0.24–0.46)	41.99	< 0.001
Age	0.08	0.01	1.08 (1.06–1.10)	41.92	< 0.001
ASMM	− 0.19	0.02	0.83 (0.80–0.86)	74.02	< 0.001
ASMM/Ht^2^	− 0.74	0.08	0.48 (0.40–0.56)	77.17	< 0.001
HGS/ASMM	− 1.25	0.18	0.29 (0.20–0.40)	51.04	< 0.001
PhA	− 0.56	0.11	0.57 (0.46–0.71)	25.35	< 0.001
Multiple model					
Intercept	1.00	1.06		0.89	0.345
Age	0.09	0.01	1.10 (1.07–1.13)	45.88	< 0.001
ASMM/Ht^2^	− 0.84	0.09	0.43 (0.36–0.52)	83.04	< 0.001
HGS/ASMM	− 1.34	0.18	0.26 (0.18–0.38)	52.10	< 0.001

Sex: men = 1, women = 0

*ASMM* appendicular skeletal muscle mass, *HGS* hand-grip strength, *Ht* height, *ASMM/Ht*^*2*^ appendicular skeletal muscle mass index, *HGS/ASMM* muscle quality index, *PhA* phase angle, *SE* standard error, *OR* odds ratio, *95% CI* confidence interval

The variables which had been found to be significantly correlated with pre-frailty in the univariate analyses were taken into account in the multiple logistic regression (Table 2). Insignificantly contributing variables were successively removed from the model. Ultimately, the following pre-frailty predictors were found to be significant: age, appendicular skeletal muscle mass index, and muscle functional quality index (Table 2). Phase angle and sex lost their statistical significance in the multiple analysis (*p* > 0.05). A stronger correlation with frailty syndrome prevalence was observed for the skeletal muscle mass and quality indices than for age. Assuming that the values for all other variables in the logit model remain constant, the odds of pre-frailty will be over twofold lower at an increase in the ASMM/Ht^2^ value by 1 kg/m^2^ (OR: 0.43, 95% CI 0.36–0.52, *p* < 0.001) and almost fourfold lower at a unit increase in HGS/ASMM (OR: 0.26, 95% CI 0.18–0.38, *p* < 0.001).

The Hosmer–Lemeshow test showed no significant differences between the expected values (pre-frail and non-frail) from the logistic model and those observed based on Fried criteria (HL_(8)_ = 9.55, *p* = 0.298). With respect to pre-frailty (*n* = 385), the sensitivity, specificity and accuracy of the model were found as 75% (287/385), 84% (528/630) and 80% (815/1015), respectively.

## Discussion

The results of our investigations confirm that the adverse changes accompanying physical frailty can be observed quite early. We have shown that in the early stage of pre-frailty, a significant decrease in skeletal muscle mass (both absolute and corrected for body size) occurs which was not so obvious as we did not observe significant differences in body mass between the pre-frail women and the non-frail women. The lowered body mass criterion for Fried’s FP was found by us to be weaker than the other criteria (strength, walking speed and physical activity). We have observed that muscle quality determines the probability of pre-frailty regardless of gender and age.

It is difficult to identify adverse muscle mass loss in older persons, especially women, since it can be masked by fatty tissue which enlarges with age. On the basis of their review of literature on correlations between body composition and frailty in elderly persons. Reinders et al. established that obesity and large waist size pose a high risk of frailty [8]. Unfortunately, the correlations between the frailty syndrome and the particular body mass components (mainly muscle mass and muscle fat infiltration) remain unclear.

Jung et al. [19] reported a correlation between frailty and a low fat-free mass index in community-dwelling older persons (Koreans aged 65 years and older (*n* = 341) under 5-year observation). The state of frailty was linked with a decrease in fat-free mass corrected for squared height. In the non-frail persons, the fat-free mass loss amounted to 0.81 ± 0.78 kg/m^2^, whereas in the pre-frail and frail persons, it was significantly greater, amounting to, respectively, 1.00 ± 0.92 kg/m^2^ and 1.35 ± 0.85 kg/m^2^. The authors assessed that in the persons with frailty, the risk of significant loss of skeletal muscle mass with age was almost three times higher than in the persons without frailty. Recently, Ishii et al. showed that physical frailty and low muscle mass significantly contributed to disability among older community-living persons [20].

Many researchers emphasize that muscle strength is the best measure of changes in muscles and it is more closely linked with physical disability and functional limitations in instrumental activities of daily living (IADL) than muscle mass [2, 20–23]. The rate of strength loss higher than the rate of muscle mass loss is due to changes in muscle composition and strength [24, 25].

We used the HGS/ASMM index and the impedance phase angle to assess the quality of skeletal muscles in the phenotype groups. The muscle strength corrected for the skeletal muscle mass is a measure of functional muscle quality and is not governed by individual variability [2, 15]. The interpretation of this index suggests that the generation of the same force at a greater ASMM means poorer quality of the skeletal muscles. In our study, skeletal muscle quality assessed through HGS/ASMM values was by nearly 10% lower in the pre-frail persons than in the non-frail persons. This means that muscle quality can be an important indicator in identifying frailty.

We found that the impedance phase angle, which is regarded as a measure of muscle cellular quality [10, 12], was significantly lower in the pre-frail persons than in the non-frail persons: by 0.2° in the men and by 0.1° in the women. No significant importance of PhA for prediction of pre-frailty suggests that the difference in muscle cellular quality observed by us was most probably due to aging (there was a 3-year difference in age between the frailty phenotype groups). This had also been observed in our previous study where in over 60-year-old persons, the 10-year difference between the age groups had generated a reduction in PhA by 0.4° in the men and by 0.3° in the women [26]. Barbosa-Silva et al. in over 70-year-old persons, they registered PhA values lower than the ones registered in persons 10 years younger: by nearly 0.8° in the men and by 0.3° in the women [27]. Moreover, the changes in PhA were found to increase with age in both gender groups [26, 27]. In their study of patients admitted to geriatric wards, diagnosed as significantly frail and with a range of comorbidities, Slee et al. [28] showed a correlation between a small phase angle and undernourishment and the frailty syndrome. They registered much lower phase angle values (4.7 ± 1.3° for the men and 4.5 ± 0.7° for the women) in comparison with our results for the pre-frail persons, which is due to differences in the frailty status and the health state. Moreover, in the study carried out by Slee et al. [28], the patients were about 13 years older; nevertheless, the range of age (62–96 years) was similar to the range of age of our subjects. Recently, Mullie et al. [29] identified the phase angle as a new biomarker of frailty in patients subjected to heart surgeries (at the age of 71 ± 8 years). They demonstrated that a smaller PhA would increase the odds of a higher degree of frailty and decline in physical fitness and mortality. They discovered significant correlations of PhA with the Fried score, the Essential Frailty Toolset score and the Short Physical Performance Battery (SPPB) score [29].

Cesari et al. [30] found strong correlations between the state of frailty and muscle quality assessed by peripheral quantitative computerized tomography (pQCT) scanning. The frail subjects had a significantly lower muscle density, a significantly smaller muscle surface area and a larger area of fat on the surface of the calf’s cross section than the non-frail subjects. Williams et al. reported that in cancer patients, skeletal muscle density was more closely correlated with the prevalence of frailty and pre-frailty than muscle mass [31].

In our study, the corrected mass of skeletal muscles and the index of their functional quality were strongly correlated with the probability of pre-frailty state, with a weaker influence of age, regardless of the gender of the subjects. These results indicate that both BIA-assessed variables can be used could be used as additional frailty identifiers. Considering the above, it would be necessary to determine reference and normalized cut-off points for the skeletal muscle mass and quality indices, which could remove the ambiguities connected with body mass decline assessment in frailty identification. This would have important implications especially for screening, where weaker tools are used to assess frailty. The availability, low cost and quickness of measurement are significant advantages of assessing ASMM by means of the bioelectrical impedance method.

The limitation of our study is the absence of frailty in the persons subjected to Fried frailty phenotype assessment. Thus, our results are applicable only to the assessment of the probability of pre-frailty. Nevertheless, we suspect that the greater reduction in muscle mass and strength in frail persons, observed by many authors, can strengthen our study’s main findings. Second, no evaluation of parameters which can have a bearing on the relationship between muscle mass, muscle function and frailty, such as protein consumption, was carried out. Also, our use of BIA, instead of the reference method, to estimate ASMM values, can be debatable, but we wanted to indicate the potential of BIA for the routine monitoring of aging, as an alternative to the often unaffordable reference methods. Moreover, the method and the ASMM predicting equation used by us have been taken into account in the latest recommendations of the European Working Group on Sarcopenia in Older People [2]. The strong point of our study is the large number of ethnically not diverse, independently living subjects.

We think that our project broadens the knowledge of frailty in elderly persons and it can be an important basis for orienting further research on improving frailty identification methods. Quick diagnosis of pre-frailty and frailty contributes to the more effective prevention of the frailty syndrome and ensures successful aging.

## Conclusion

This study has indicated that the easily available and inexpensive method of BIA can be used to preventively monitor changes not only in the mass of skeletal muscles, but also in their quality, which is particularly important in the case of pre-frail older persons. The presented results of the study confirm that the skeletal muscle quantity and quality indices based on BIA estimates can facilitate the assessment of the state of frailty in routine geriatric care.
